# IL3RA-Targeting Antibody–Drug Conjugate BAY-943 with a Kinesin Spindle Protein Inhibitor Payload Shows Efficacy in Preclinical Models of Hematologic Malignancies

**DOI:** 10.3390/cancers12113464

**Published:** 2020-11-20

**Authors:** Dennis Kirchhoff, Beatrix Stelte-Ludwig, Hans-Georg Lerchen, Antje Margret Wengner, Oliver von Ahsen, Pascale Buchmann, Stephan Märsch, Christoph Mahlert, Simone Greven, Lisa Dietz, Michael Erkelenz, Ruprecht Zierz, Sandra Johanssen, Dominik Mumberg, Anette Sommer

**Affiliations:** 1 Bayer AG, Pharmaceuticals, Research & Development, 13342 Berlin, Germany; antje.wengner@bayer.com (A.M.W.); oliver.vonahsen@bayer.com (O.v.A.); michael.erkelenz@bayer.com (M.E.); ruprecht.zierz@bayer.com (R.Z.); sandra.johanssen@bayer.com (S.J.); dominik.mumberg@bayer.com (D.M.); 2 Bayer AG, Pharmaceuticals, Research & Development, 42096 Wuppertal, Germany; beatrix.stelte-ludwig@bayer.com (B.S.-L.); hans-georg.lerchen@bayer.com (H.-G.L.); pascale.buchmann@bayer.com (P.B.); stephanmaersch@hotmail.com (S.M.); christoph.mahlert@bayer.com (C.M.); simone.greven@bayer.com (S.G.); lisa.dietz@bayer.com (L.D.)

**Keywords:** acute myeloid leukemia, antibody-drug conjugate, CD123, IL3RA, kinesin spindle protein inhibitor

## Abstract

**Simple Summary:**

IL3RA (alpha subunit of the interleukin 3 receptor) is a cell membrane protein frequently expressed in acute myeloid leukemia (AML) and Hodgkin lymphoma; therefore, it is a promising therapeutic target for cancer treatment. Here, we introduce BAY-943, a novel IL3RA-targeting antibody–drug conjugate that shows potent and selective efficacy in IL3RA-positive AML and Hodgkin lymphoma cell lines. In IL3RA-positive AML mouse models, BAY-943 improved survival and reduced tumor burden. Impressively, treatment with BAY-943 induced complete tumor remission in 12 out of 13 mice in an IL3RA-positive HL model. BAY-943 showed a favorable safety profile without any signs of toxicity in rats and monkeys. Overall, these preclinical results support the further development of BAY-943 for the treatment of IL3RA-positive hematologic malignancies.

**Abstract:**

IL3RA (CD123) is the alpha subunit of the interleukin 3 (IL-3) receptor, which regulates the proliferation, survival, and differentiation of hematopoietic cells. IL3RA is frequently expressed in acute myeloid leukemia (AML) and classical Hodgkin lymphoma (HL), presenting an opportunity to treat AML and HL with an IL3RA-directed antibody–drug conjugate (ADC). Here, we describe BAY-943 (IL3RA-ADC), a novel IL3RA-targeting ADC consisting of a humanized anti-IL3RA antibody conjugated to a potent proprietary kinesin spindle protein inhibitor (KSPi). In vitro, IL3RA-ADC showed potent and selective antiproliferative efficacy in a panel of IL3RA-expressing AML and HL cell lines. In vivo, IL3RA-ADC improved survival and reduced tumor burden in IL3RA-positive human AML cell line-derived (MOLM-13 and MV-4-11) as well as in patient-derived xenograft (PDX) models (AM7577 and AML11655) in mice. Furthermore, IL3RA-ADC induced complete tumor remission in 12 out of 13 mice in an IL3RA-positive HL cell line-derived xenograft model (HDLM-2). IL3RA-ADC was well-tolerated and showed no signs of thrombocytopenia, neutropenia, or liver toxicity in rats, or in cynomolgus monkeys when dosed up to 20 mg/kg. Overall, the preclinical results support the further development of BAY-943 as an innovative approach for the treatment of IL3RA-positive hematologic malignancies.

## 1. Introduction

Interleukin 3 receptor subunit alpha (IL3RA; also known as CD123) is the α subunit of the heterodimeric IL-3 receptor. Together with the β subunit, it forms a functional high-affinity receptor for IL-3 [1,2,3]. IL-3 is a pleiotropic cytokine that is mainly produced by activated T lymphocytes, and it regulates the function and production of hematopoietic and immune cells [4]. IL3RA is expressed at high levels in ≈80% of acute myeloid leukemias (AML) [1,2,5], 59–100% of classical Hodgkin lymphomas (cHL), and the majority of blastic plasmacytoid dendritic cell neoplasms (BPDCN) [6,7,8,9,10]. It is also expressed by close to 100% of myelodysplastic syndrome (MDS) patients, but the expression intensity may vary [11,12,13,14]. Importantly, IL3RA overexpression on AML blasts has been associated with an increased number of leukemic blast cells at diagnosis and with a negative prognosis [15]. IL3RA is also expressed in basophils and plasmacytoid dendritic cells [5,16].

Several studies have indicated that IL-3 and its receptor play important roles in the progression of AML [3,17], and indeed, experiments with a monoclonal antibody that blocks the binding of IL-3 to IL3RA have shown increased survival in AML mouse models [18]. Characterization of hematologic malignancies has demonstrated increased IL3RA expression in CD34^+^CD38^−^ AML blasts as compared to expression in normal cells. Furthermore, these IL3RA-overexpressing cells have been shown to be able to initiate and maintain the leukemic process in immuno-deficient mice and thus act as leukemic stem cells [3,19]. Consequently, IL3RA has been shown to be a very useful biomarker for the detection of minimal residual disease, thereby predicting relapse in AML patients [20,21]. Taken together, these results suggest that IL3RA is a very attractive target for an antibody–drug conjugate (ADC) approach for the treatment of AML and other IL3RA-positive hematologic malignancies [10].

Here, we exploited a novel pyrrole subclass payload that potently inhibits the kinesin spindle protein (KSP/KIF11/Eg5) in biochemical and cellular assays to develop an ADC to target IL3RA on cancer cells [22,23,24,25]. KSP is a motor protein responsible for an essential event in mitosis, the segregation of duplicated centrosomes during spindle formation in the G2/M phase of the cell cycle, and therefore, it is required for productive cell divisions [26]. High expression of KSP in hematologic indications such as AML blasts and diffuse large B-cell lymphoma (DLBCL) [27] and in solid cancers such as breast, bladder, and pancreatic cancer has been linked to poorer prognosis [28], and thus, KSP presents an attractive target for cancer treatment.

KSP is active in all proliferating cells and therefore, KSP inhibitors (KSPi) representing various structural classes have resulted in neutropenia, mucositis, and stomatitis in clinical trials [28,29,30,31,32]. However, ADCs that combine a cancer cell-targeting antibody and a cytotoxic payload via a linker can deliver a cytotoxic payload specifically to target-expressing cancer cells. This approach could protect healthy tissue from exposure to the cytotoxic compound, thus decreasing overall side effects especially on highly proliferative tissues, thereby expanding the therapeutic window.

To generate the IL3RA-ADC BAY-943, a non-cell-permeable KSPi was conjugated randomly to the lysine residues of a humanized derivative of the anti-IL3RA antibody 7G3 [33], TPP-9476, via a novel protease-cleavable peptide linker [24]. IL3RA-ADC was efficacious in IL3RA-positive AML and HL cell lines in vitro, as well as in IL3RA-expressing AML and HL cell line and patient-derived xenograft (PDX) models in vivo. IL3RA-ADC was well-tolerated in the mouse, rat, and cynomolgus monkey. No signs of neutropenia, mucositis, or stomatitis, the typical side effects of small molecule KSPis, were observed in safety studies performed in rat and cynomolgus monkey. Taken together, these data support the further development of the compound as a novel therapy option for patients with AML or other hematologic malignancies expressing IL3RA.

## 2. Results

### 2.1. Characterization of the IL3RA-Targeting Antibody TPP-9476 and IL3RA-ADC BAY-943

**Figure 1:**
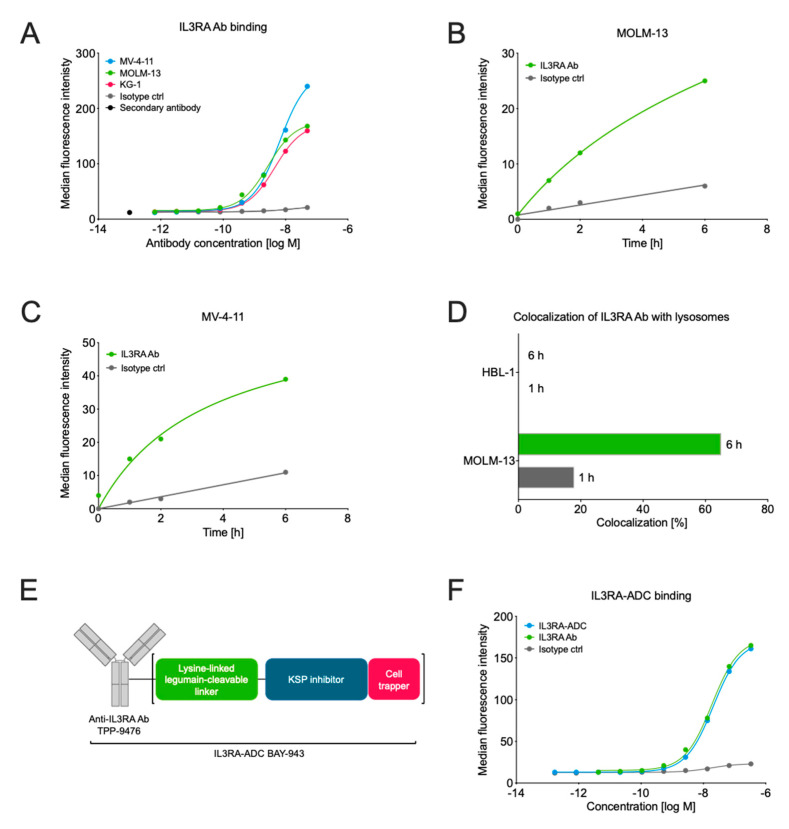
Characterization of the interleukin 3 receptor subunit alpha (IL3RA) antibody TPP-9476 and schematic representation of the IL3RA antibody–drug conjugate (ADC) BAY-943. (**A**) The binding of the IL3RA-targeting antibody (IL3RA Ab) TPP-9476 to IL3RA-positive hematologic cell lines as determined by flow cytometry. The obtained EC_50_ values were 2.73 × 10^−9^ M for MOLM-13, 6.53 × 10^−9^ M for MV-4-11, and 4.54 × 10^−9^ M for KG-1 cells. (**B**,**C**) The internalization of the IL3RA Ab TPP-9476 and an isotype control antibody into IL3RA-positive MOLM-13 (**B**) and MV-4-11 (**C**) cells as determined by flow cytometry-based imaging. (**D**) The colocalization of the IL3RA Ab TPP-9476 in lysosomes in the IL3RA-positive MOLM-13 and IL3RA-negative HBL-1 cells. (**E**) Schematic representation of the IL3RA-ADC BAY-943. TPP-9476 represents the IL3RA-Ab. The “cell trapper” functionality indicates a non-cell-permeable payload metabolite that enables maximal retention in target cells after cleavage. (**F**) The binding of the IL3RA-ADC BAY-943 to IL3RA-positive MOLM-13 cells as determined by flow cytometry. The obtained EC_50_ values were 20.4 × 10^−9^ M for ILRA3A-ADC and 18.7 × 10^−9^ M for ILRA3A Ab, respectively.

The binding affinity of the IL3RA-targeting antibody TPP-9476 (IL3RA-Ab) to human and cynomolgus monkey IL3RA was assessed by surface plasmon resonance (SPR) and flow cytometry. IL3RA-Ab showed high affinity to both the human and the cynomolgus monkey IL3RA protein with dissociation constants (KD) of 11 nmol/L and 16 nmol/L, respectively, as determined by SPR. No binding to murine IL3RA was observed. Furthermore, IL3RA-Ab bound specifically to the IL3RA-expressing human hematologic cancer cell lines MOLM-13, MV-4-11, and KG-1 as determined by flow cytometry (Figure 1A).

As the prerequisite for ADC activity is to effectively deliver the cytotoxic payload into the cells, we next studied the ability of IL3RA-Ab to internalize upon target binding. The fluorescently labeled IL3RA-Ab showed highly specific, target-dependent internalization in the IL3RA-positive MOLM-13 and MV-4-11 cell lines with >3.5-fold enhancement as compared with the non-specific internalization of the isotype control antibody (Figure 1B,C). In flow cytometry-based imaging, IL3RA-Ab showed lysosomal colocalization in the IL3RA-positive MOLM-13 but not in the IL3RA-negative HBL-1 cell line (Figure 1D). The lysosomal colocalization of IL3RA-Ab indicates that when incorporated into an ADC, it allows the release of the payload metabolite. This can occur via the cleavage of the linker by a lysosomal protease that is active at acidic pH (such as legumain) and/or by proteolytic degradation of the antibody.

Since IL3RA-Ab demonstrated the essential properties of an effective ADC antibody, we conjugated a non-cell permeable KSPi to the lysine residues of the IL3RA-Ab TPP-9476 via a novel legumain-cleavable peptide linker [24] to produce the IL3RA-targeting ADC BAY-943 (IL3RA-ADC; Figure 1E). IL3RA-ADC showed high stability in human plasma (Supplementary Figure S1C) and a comparable binding affinity to the IL3RA-Ab (half-maximal effective concentration, EC_50_ 18–21 nmol/L in MOLM-13 cells; Figure 1F), indicating that the attachment of the KSPi payload linker does not impact the binding affinity of the ADC antibody moiety. Furthermore, the active payload metabolite of IL3RA-ADC, BAY-716, showed poor permeability across Caco-2 cells (apparent permeability, Papp A-B = 1.8 nm/s, Papp B-A = 2.7 nm/s) with an efflux ratio (Papp B-A/Papp A-B) of 1.6, indicating that no active efflux takes place in Caco-2 cells. The poor permeability from B-A in Caco-2 cells indicates a long residence time after intracellular release of the active KSPi metabolite BAY-716 in tumor cells. As Caco-2 cells express the efflux transporter P-gP (P-glycoprotein), it also suggests that BAY-716 is a poor substrate for the efflux transporter P-gP.

### 2.2. IL3RA-ADC Shows Potent and Selective Efficacy In Vitro

The in vitro cytotoxicity of the IL3RA-ADC BAY-943 was assessed in a panel of human tumor cell lines with different IL3RA expression levels (Table 1). IL3RA-ADC demonstrated potent antiproliferative activity with half-maximal inhibitory concentration (IC_50_) values at the subnanomolar to nanomolar range in the IL3RA-positive AML (MV-4-11, MOLM-13) and HL (HDLM-2, L-428) derived cell lines, whereas little activity was observed in the tumor cell lines with low levels of or negative for IL3RA membrane expression (NCI-H292, HT). Moreover, in IL3RA-positive AML and HL cell lines, a 10 to 100-fold higher sensitivity to IL3RA-ADC compared to the non-targeted isotype control ADC was observed (Table 2), demonstrating that the activity of IL3RA-ADC is target-dependent. Furthermore, IL3RA-ADC was found to induce apoptosis specifically in IL3RA-positive cells, as demonstrated by caspase 3/7 activation in MV-4-11 with an EC_50_ of 4.33 nmol/L, but not in the IL3RA-negative MDA-MB-231 cells (EC_50_ > 300 nmol/L; Supplementary Figure S2), further supporting the selectivity of IL3RA-ADC.

### 2.3. IL3RA-ADC Improves Survival in the MOLM-13 and MV-4-11 Xenograft Models

**Figure 2:**
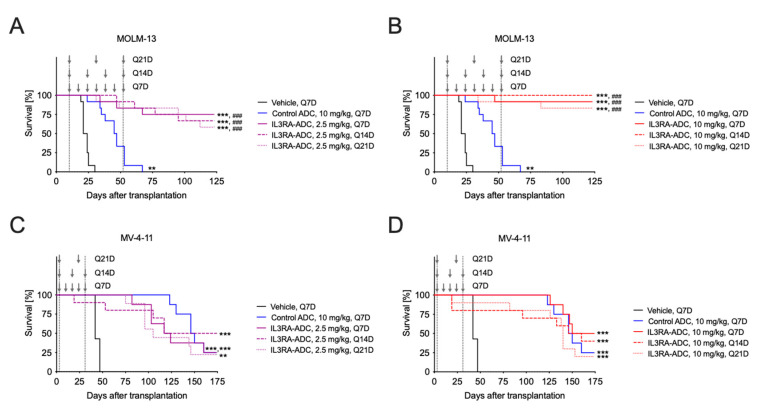
Antitumor efficacy of the IL3RA-ADC BAY-943 in the systemic MOLM-13 and MV-4-11 leukemia xenograft models. A-B. Kaplan–Meier survival plots of mice transplanted with the MOLM-13 human acute myeloid leukemia (AML) cells and treated intravenously (i.v.) with the isotype control ADC (10 mg/kg, Q7D) or IL3RA-ADC at 2.5 mg/kg (**A**) or 10 mg/kg (**B**); Q7D, Q14D, or Q21D. C-D. Kaplan–Meier survival plots of mice transplanted with the MV-4-11 human biphenotypic leukemia cells and treated i.v. with the isotype control ADC (10 mg/kg, Q7D) or IL3RA-ADC at 2.5 mg/kg (**C**) or 10 mg/kg (**D**); Q7D, Q14D or Q21D. The vertical dashed gray lines delineate the treatment period, and the arrows indicate time of treatment. Data were analyzed using the Cox proportional hazards model and corrected for family-wise error rate using Sidak’s method. Asterisks and hashtags indicate statistical significance in comparison to the vehicle (** *p* < 0.01, *** *p* < 0.001) or isotype control ADC (### *p* < 0.001).

The antitumor efficacy of the IL3RA-ADC BAY-943 was tested in two IL3RA-positive, systemic (intravenous transplantation) cell line-derived xenografts: MOLM-13 human AML and MV-4-11 human biphenotypic leukemia models in mice. Both the MOLM-13 and MV-4-11 cell lines harbor *FLT3-ITD* mutations shown to be associated with an unfavorable prognosis in AML [34]. The median survival time (MST) for the vehicle and isotype control ADC was 22.5 and 46, respectively (Figure 2). By contrast, in the MOLM-13 model, 80–100% of the mice treated with 10 mg/kg IL3RA-ADC survived without signs of leukemia until day 123, when the study was terminated (Figure 2B), while all mice treated with the isotype control ADC were sacrificed due to signs of disease by day 67 after tumor cell inoculation. In the MV-4-11 model, the administration of IL3RA-ADC once weekly (Q7D), every two weeks (Q14D), or every three weeks (Q21D) resulted in a potent and sustained antitumor effect with MSTs of 120.5, 145.5, and 105 at the IL3RA-ADC dose of 2.5 mg/kg (Figure 2C) and 162, 153, and 140 days at the IL3RA-ADC dose of 10 mg/kg, respectively (Figure 2D). The MST for the vehicle and the isotype control ADC was 48 and 148, respectively. No significant differences in efficacy between the tested treatment schedules were observed in either of the models.

In the vehicle and the isotype control ADC groups, nearly all mice had symptoms of leukemia, i.e., splenomegaly and paralysis of hind limbs. In addition, the mean body weights decreased in these treatment groups, indicating that the mice suffered from leukemia (Supplementary Figure S3). However, no treatment-related side effects or abnormalities were observed during the study or at gross necropsy in the IL3RA-ADC-treated groups.

The antitumor efficacy of IL3RA-ADC was also tested in the subcutaneous MOLM-13 and MV-4-11 xenograft models in mice. Repetitive dosing with IL3RA-ADC resulted in a significant suppression of tumor growth in both models compared to the isotype control ADC, while the standard-of-care cytarabine showed no activity in these models (Supplementary Figure S4). Furthermore, in the MV-4-11 model, treatment with the unconjugated IL3RA-Ab TPP-9476 at 5 mg/kg, Q7D×2 showed no tumor growth inhibition (Supplementary Figure S4C,D), indicating that the antitumor activity of IL3RA-ADC is conveyed by the targeted delivery of the KSPi payload and not the IL3RA-Ab.

### 2.4. IL3RA-ADC Suppresses Tumor Burden and Improves Survival in Systemic AM7577 and AML11655 PDX Models

The efficacy of IL3RA-ADC was further evaluated in the systemic AM7577 and AML11655 patient-derived AML xenograft models in mice. These PDX models showed high IL3RA protein expression (Supplementary Figure S5) and harbor a typical AML genotype with mutations in genes including *NMP1*, *FLT3-ITD*, *IDH1*, *IDH2*, and *DNMT3A* (Supplementary Table S1).

**Figure 3:**
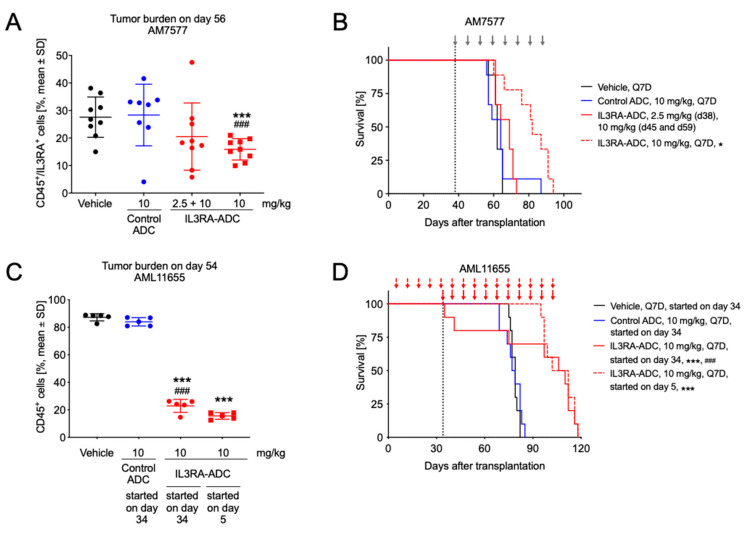
Antitumor efficacy of the IL3RA-ADC BAY-943 in the patient-derived AM7577 and AML11655 AML xenograft models. (**A**) Tumor burden on day 56 in mice transplanted with AM7577 cells and treated i.p. with the isotype control ADC (10 mg/kg, Q7D) and IL3RA-ADC (2.5 + 10 mg/kg or 10 mg/kg, Q7D). In the 2.5 + 10 mg/kg IL3RA-ADC treatment group, the first dose was 2.5 mg/kg (day 38) and the two subsequent doses (on days 45 and 59) 10 mg/kg. (**B**) Kaplan–Meier survival plots of mice described in panel A. Treatment days in all groups except for the 2.5 + 10 mg/kg IL3RA-ADC treatment group are indicated with gray arrows. (**C**) Tumor burden on day 54 in mice transplanted with AML11655 cells. Intraperitoneal treatments with the isotype control ADC (10 mg/kg, Q7D) were initiated on day 34 and with IL3RA-ADC (10 mg/kg, Q7D) on day 5 (preventive setting) or 34 (therapeutic setting). (**D**) Kaplan–Meier survival plots of mice described in panel C. Treatment days are indicated with red arrows. The data were analyzed using the Cox proportional-hazards model and corrected for family-wise error rate using Sidak’s method. Asterisks and hashtags indicate statistical significance in comparison to vehicle (* *p* < 0.05, *** *p* < 0.001) and the isotype control ADC (### *p* < 0.001).

In the AM7577 PDX model, IL3RA-ADC administered at 10 mg/kg intraperitoneally (i.p.) reduced tumor burden compared to the vehicle or isotype control ADC, as indicated by a decreased number of human CD45 (hCD45)/human IL3RA (hIL3RA)-positive cells in blood on day 56 (both *p* < 0.001; Figure 3A). Furthermore, treatment with IL3RA-ADC resulted in improved survival with the MST of 69 days at the dose of 2.5 + 10 mg/kg and 82 days at the dose of 10 mg/kg. The MST for the vehicle and the isotype control ADC was 62 and 64 days, respectively (Figure 3B).

In the AML11655 mouse xenograft model, IL3RA-ADC was administered i.p. using either a preventive (treatment started on day 5) or a therapeutic (treatment started on day 34) setting. IL3RA-ADC administered at 10 mg/kg inhibited the growth of IL3RA-positive AML cells, as indicated by temporarily reduced numbers of hCD45-positive cells in blood compared to vehicle or isotype control ADC in both settings (Figure 3C,D; all *p* < 0.001). In addition, treatment with IL3RA-ADC using either the preventive or therapeutic setting resulted in prolonged MSTs of 107 or 108 days, respectively (Figure 3D). The MST for the vehicle and the isotype control ADC was 79 and 78 days, respectively.

### 2.5. IL3RA-ADC Demonstrates Antitumor Efficacy in Subcutaneous HDLM-2 Hodgkin Lymphoma Xenograft Model

**Figure 4:**
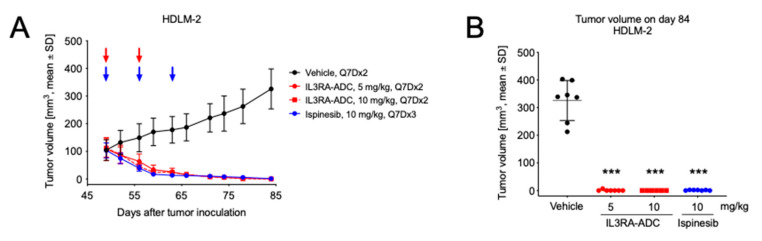
Antitumor efficacy of the IL3RA-ADC BAY-943 in the subcutaneous HDLM-2 Hodgkin lymphoma xenograft model. Mice were transplanted with HDLM-2 cells and treatments with IL3RA-ADC (5 or 10 mg/kg, Q7D×2, i.p.) or ispinesib (10 mg/kg, Q7D×3, i.v.) were initiated on day 49. (**A**) Tumor growth curves. ADC treatment days are indicated with red arrows and ispinesib administration is indicated with blue arrows. (**B**) Tumor volume on day 84. Statistical analyses were performed using a linear mixed-effects model with random intercepts and slopes for each subject (n = 6–7). Mean comparisons between the treatment and control groups were performed using the estimated linear mixed-effects model and corrected for family-wise error rate using Sidak’s method. Asterisks indicate statistical significance in comparison to vehicle (*** *p* < 0.001).

Finally, the antitumor efficacy of the IL3RA-ADC BAY-943 was tested in a subcutaneous HDLM-2 Hodgkin lymphoma xenograft model in mice. This model showed a high IL3RA antigen density with ≈74,300 anti-IL3RA antibodies bound per cell (Table S1), which is in line with the literature [8]. In the HDLM-2 model, the i.p. injection of IL3RA-ADC at 5 or 10 mg/kg resulted in a strong reduction of tumor growth compared to the vehicle (both *p* < 0.001; Figure 4). This effect was comparable with the clinically studied KSPi ispinesib administered at 10 mg/kg in the same model. In the two IL3RA-ADC treatment groups, total tumor eradication was observed in twelve mice out of thirteen (92%) at the end of the study.

### 2.6. IL3RA-ADC Is Well-Tolerated

The safety, including possible changes in the hematologic cell populations, of IL3RA-ADC was evaluated in the cynomolgus monkey in two range-finding studies with single or repeated dosing. IL3RA-ADC was well-tolerated without adverse events typically observed with ADCs containing other payload classes, such as thrombocytopenia, neutropenia, or signs of liver toxicity. In addition, mucositis, a dose-limiting toxicity for small molecule KSP inhibitors in clinical studies [35], was not observed. A single dose of IL3RA-ADC up to 20 mg/kg or three repeated doses of IL3RA-ADC up to 10 mg/kg given every three weeks resulted in a transient reduction of IL3RA-positive basophils and plasmacytoid dendritic cells (pDCs), indicating targeting of the IL3RA-ADC to antigen-positive cells (data not shown). Furthermore, the administration of IL3RA-ADC showed no obvious changes in the percentage of the CD34^+^Lin^−^ bone marrow cell population containing the hematopoietic stem cells, as analyzed by flow cytometry (data not shown).

The effect of the toxophore metabolite BAY-716 was also analyzed after a single dose of 0.25 mg/kg in rats (data not shown). No laboratory or histopathology findings were observed, indicating that this non-cell-permeable toxophore metabolite does not induce toxic effects thereby underlining the good safety profile of IL3RA-ADC.

## 3. Discussion

Despite the recent progress in the treatment of AML, clinical outcomes have improved only minimally over the past three decades. Therefore, novel therapeutic agents with a larger therapeutic window and a favorable tolerability profile are urgently needed to improve the therapeutic outcome for AML patients. Increasing evidence indicates that IL3RA is highly expressed in leukemic stem cells but not in normal hematopoietic stem cells, and it associates in AML with treatment response, minimal residual disease detection, and prognosis [3,15,17]. Consequently, several IL3RA-targeting approaches, such as an anti-IL3RA antibody enhanced for antibody-dependent cell-mediated cytotoxicity, anti-IL3RA-ADCs with highly potent payloads of the pyrrolobenzodiazepine (PBD) or indolinobenzodiazepine pseudodimer (IGN) class, various bispecific T cell recruiting antibodies, or chimeric antigen receptor T cell (CAR-T) therapies are currently under preclinical or clinical development [10,28,36,37,38,39].

Here, we explored a novel concept to improve the therapeutic window and safety of KSP inhibition by targeting a non-cell-permeable KSP inhibitor as ADC to AML cells, and thereby, sparing fast-dividing healthy cells from KSP inhibition. This provides a payload with a novel mode of action and would be a new therapeutic option for the treatment of IL3RA-positive malignancies. The investigated IL3RA-targeting ADC (BAY-943, IL3RA-ADC) consists of a humanized anti-IL3RA antibody conjugated with a stable lysine linkage to a potent proprietary KSPi via a protease-cleavable linker, producing a non-cell-permeable payload metabolite.

KSP is an ATP-dependent plus-end directed motor protein, which generates force and moves along microtubules, and it is involved in the separation of the centrosomes, the generation of the bipolar spindle, and thereby plays an important role in mitosis [26]. The inhibition of KSP with small molecules such as monastrol or small interfering RNA (siRNA)-mediated knockdown results in the formation of monopolar spindles (termed a “monoaster”), which lead to aberrant mitotic arrest and apoptosis [30,40]. Thus, KSP presents a convincing target for the development of an anti-mitotic approach for cancer treatment. Accordingly, several allosteric KSP inhibitors such as ispinesib, litronesib, and filanesib (ARRY-520) have been or are in clinical trials [35,41,42,43,44]. Filanesib has also been explored in a Phase I clinical trial in AML [43], and clinical studies are ongoing in relapsed refractory multiple myeloma (rrMM). The antitumor activity of filanesib has previously been shown in AML cells in vitro and in xenograft mouse models [27]. The most common side effects of KSP inhibitors with different chemical scaffolds are neutropenia, mucositis, and stomatitis [35]. This has been explained by the inhibition of KSP in highly proliferative cells such as neutrophils and cells lining the mucosa and the stoma, respectively, thus limiting their therapeutic efficacy due to a small therapeutic window.

Antibody–drug conjugates (ADCs) are one solution that has been proposed to mitigate the toxic side effects of anti-mitotic therapies and to broaden their therapeutic window. In fact, currently more than 60 ADCs against multiple targets in solid and hematologic tumors are in clinical trials [45,46], and eight ADCs have meanwhile been approved [47]. The payload classes currently used are confined to microtubule destabilizers (e.g., auristatin, dolastatin, maytansinoid, tubulysine), DNA interacting agents (e.g., calicheamicin, duocarmycin, PBD, IGN), and topoisomerase inhibitors (e.g., camptothecin derivative SN-38, exatecan). Many of these permeable payloads and/or highly potent DNA-interacting payloads have safety issues and therefore result in an insufficient therapeutic window. For example, the clinical trials for the CD33-targeting SGN-CD33A and the IL3RA-targeting SGN-CD123A [38,39] both with a PBD payload were terminated in 2017 and 2018, respectively. Recently, the first IL3RA-targeting therapy was approved for BPDCN [9]. However, the fusion protein tagraxofusp-erzs (formerly called SL-401), which consists of the ligand IL3 fused to a truncated diphtheria toxin, has been reported to cause capillary leak syndrome as a common side effect in more than 55% of patients [10]. This further underlines that efficacious therapies with acceptable safety profiles are still urgently required for targeting IL3RA-positive malignancies.

The IL3RA-ADC BAY-943 demonstrated the capability of delivering a novel cytotoxic payload to IL3RA-positive cells. The IL3RA antibody TPP-9476 to which the KSP inhibitor payload is linked via a legumain-cleavable peptide linker showed high binding affinity and specificity to IL3RA and bound specifically to IL3RA-expressing human AML and HL cell lines. The IL3RA antibody internalized into the lysosomes of IL3RA-positive MOLM-13 and MV-4-11 AML cell lines, and IL3RA-ADC demonstrated high cytotoxic potency in IL3RA-positive MV-4-11 and MOLM-13 AML and HDLM-2 and L-428 HL derived cell lines. Furthermore, in the IL3RA-positive cell lines tested, IL3RA-ADC showed 10 to 1000-fold cytotoxicity compared with the isotype control ADC, indicating high target selectivity. The less prominent selectivity observed in MOLM-13 and THP-1 cells may be explained by a non-specific uptake of the isotype control ADC to AML cells differentiated along the macrophage lineage.

In an in vivo setting, IL3RA-ADC administered at 10 mg/kg increased survival in both the IL3RA-positive MOLM-13 and MV-4-11 cell line-derived and IL3RA-positive AM7577 and AML11655 patient-derived AML xenograft models harboring molecular alterations associated with poor prognosis in AML. The increased survival was accompanied by a reduction in the growth of IL3RA-positive AML cells and tumor size in the systemic and subcutaneous models, respectively. In the IL3RA-positive HDLM-2 subcutaneous Hodgkin lymphoma model, IL3RA-ADC treatment also resulted in significant antitumor efficacy with most animals being tumor-free at the end of the study.

The body weights of the animals decreased over the course of the study, indicating that they suffered from leukemia. However, no IL3RA-ADC treatment-related body weight losses were observed, suggesting good tolerability (Supplementary Figures S3 and S6), particularly in comparison to the small molecule KSPi ispinesib, which induced a transient body weight loss in mice after the second treatment (Supplementary Figure S6C). The treatments with IL3RA-ADC in the HDLM-2 subcutaneous Hodgkin lymphoma model were also well-tolerated.

The good tolerability of the IL3RA-ADC was confirmed by repeated dose safety and immunotoxicity assessments in the cynomolgus monkey with no changes in the portion of the CD34^+^Lin^−^ cell population, and transient decreases in basophils and IL3RA-positive basophils. Importantly, liver toxicity, thrombocytopenia, and neutropenia, which are frequently observed with ADCs in the clinic and in cynomolgus monkey preclinical studies [48,49], were not apparent, which was most likely due to the fact that the IL3RA-ADC toxophore metabolite is non-cell permeable. In addition, neutropenia and mucositis, which were the dose-limiting toxicities for small molecule KSP inhibitors in the clinic, were not observed. Furthermore, the IL3RA-ADC metabolite BAY-716 showed poor permeability across Caco-2 cells, indicating that the metabolite is trapped in tumor cells after its intracellular release. This “cell trapper” functionality enables a long-lasting exposure and at the same time potentially reduces off-target toxicities through the low permeability of KSPi into normal cells.

## 4. Materials and Methods

### 4.1. Cell Lines

Cell lines were acquired from DSMZ (German Collection of Microorganisms and Cell Cultures GmbH; Braunschweig, Germany) unless otherwise noted and cultured according to the provider’s instructions. Human MDA-MB-231 breast cancer, NCI-H292 non-small cell lung cancer, MV-4-11 and THP-1 acute monocytic leukemia, KG-1 acute myelogenous leukemia, Rec-1 mantle cell lymphoma, and Ramos Burkitt’s lymphoma cells were obtained from ATCC (American Type Culture Collection; Manassas, VA, USA). Human OVCAR-8 ovarian cancer cells were acquired from the NCI-60 Human Tumor Cell Line Panel (National Cancer Institute, Rockville, MD, USA). The human diffuse B cell lymphoma cell line HBL-1 was obtained from Dr. Georg Lenz (Charité Universitätsklinikum, Berlin, Germany) and cultivated in RPMI 1640 supplemented with 10% fetal calf serum (FCS). The human Hodgkin lymphoma cell line L-428 (source not known) was cultivated in RPMI 1640 supplemented with 10% FCS. Cancer cell lines were obtained between 2002 and 2012, authenticated using short tandem repeat DNA fingerprinting at DSMZ (Table 1), and subjected frequently to mycoplasma testing.

### 4.2. Compounds

The anti-IL3RA antibodies TPP-9476 and TPP-8988 (recognizes a different epitope in the extracellular domain of IL3RA than TPP-9476) and the isotype control antibody TPP-754, the IL3RA-ADC BAY-943, the isotype control ADC BAY-229 (with TPP-754), the non-cell-permeable toxophore metabolite BAY-716 (active toxophore metabolite of IL3RA-ADC), and the cell-permeable small molecule KSPi BAY-331 were manufactured at Bayer AG. Ispinesib (SYNT1009) was purchased from Syncom (a contract research organization in organic chemistry, www.syncom.eu; Groningen, the Netherlands), cytarabine (HT0476) from Accord Healthcare GmbH (Neutraubling, Germany), and staurosporine (#S4400) from Sigma-Aldrich (Saint Louis, MO, USA).

The IL3RA-specific hIgG1 antibody (IL3RA-Ab, TPP-9476) was generated by humanization of the murine anti-IL3RA antibody 7G3 [33] as described in Lerchen et al. [22]. During a protein engineering process, which is meant to bring the amino acid sequence as close as possible to the next human germline [50], multiple variants were tested. The final version, TPP-9476, comprises several amino acid exchanges in the light and heavy chain that resulted in enhanced internalization.

The generation and characterization of the IL3RA-Ab is described in the Supplementary Methods. The IL3RA-targeting ADC BAY-943 (IL3RA-ADC) was generated by conjugating the KSPi to the lysine residues of IL3RA-Ab via a protease-cleavable linker [24]. The characterization of IL3RA-ADC is described in the Supplementary Methods. In the in vivo efficacy studies, IL3RA-ADC BAY-943 with a drug-to-antibody ratio (DAR) of 6.3 as determined by mass spectrometry was used. At a DAR of 6.3, no aggregation of the IL3RA-ADC was observed (Supplementary Figure S1)

### 4.3. Internalization and Lysosomal Colocalization of IL3RA-Ab

Internalization and colocalization experiments were performed in MOLM-13 and MV-4-11 AML cell lines using flow cytometry-based imaging. The IL3RA-specific antibody TPP-9476, IL3RA-ADC BAY-943, corresponding isotype control antibody TPP-754, and isotype control ADC BAY-229 were lysine-conjugated with a ten-fold molar excess of CypHer 5E mono NHS ester (GE Healthcare, Chicago, IL, USA) at pH 8.3. The reaction mixture was purified by chromatography (PD10 desalting column, GE Healthcare, Chicago, IL, USA), followed by centrifugation (Vivaspin 500, Sartorius Stedim Biotech, Aubagne, France). Alexa 488 (Jackson ImmunoResearch, West Grove, PA, USA) was utilized as a constitutive dye. The fluorescence was measured using the Amnis^®^ FlowSight^®^ or the Guava easyCyte^TM^ flow cytometers (Luminex Corporation, Austin, TX, USA) and analyzed using the IDEAS^®^ software or the guavaSoft 2.6 software (Luminex Corporation, Austin, TX, USA).

For the internalization assay, the tumor cells (5 × 10^4^/well) were incubated with the labeled antibodies (10 µg/mL) at 37 °C, 5% CO_2_ for 0, 1, 2, and 6 h. The fluorescence was measured using the Amnis^®^ FlowSight^®^ or the Guava easyCyte^TM^ flow cytometers and analyzed using the IDEAS^®^ or the guavaSoft 2.6 software. The kinetics of the internalization were determined based on the analysis of the median fluorescence intensity (MFI) over time.

For the colocalization studies, MOLM-13 and MV-4-11 tumor cells (5 × 10^4^/well) were incubated with the labeled antibodies (20 µg/mL) at 37 °C, 5% CO_2_ for 0 h, 0.5 h, 2 h, and 6 h. The lysosomal compartment marker CytoPainter LysoGreen (1:2000; Abcam, Cambridge, UK) was added 30 min before the end of the incubation period. After incubation, the cells were washed and resuspended in ice-cold FACS (fluorescence-activated cell sorting) buffer consisting of phosphate-buffered saline (PBS) and 3% FCS. The lysosomal colocalization was analyzed with FACS image analysis using the IDEAS^®^ software.

The assessment of the stability of the IL3RA-ADC BAY-943 in human plasma and the permeability of the KSPi toxophore metabolite BAY-716 in Caco-2 cells are described in the Supplementary Methods.

### 4.4. In Vitro Cytotoxicity of IL3RA-ADC

The antiproliferative activity of IL3RA-ADC was determined in a panel of human tumor cell lines using the CellTiter-Glo^®^ assay (Promega Madison, WI, USA). Cells (2000–5000 cells/well) were incubated at 37 °C, 5% CO_2_ for 24 h and the compounds were added at concentrations of 3 × 10^−11^–3 × 10^−7^ M (or 3 × 10^−12^–3 × 10^−8^ M, depending on the cell line tested) in triplicates. Cell viability was determined at the beginning (day 0) and after 72 h incubation in the presence or absence of ADCs. The IC_50_ of the growth inhibition was calculated in comparison to day 0. The IL3RA antigen density was determined with the QIFI (Dako, Glostrup, Denmark) quantitative flow cytometry assay using the murine anti-IL3RA antibody clone 7G3 (Becton Dickinson, Franklin Lakes, NJ, USA).

### 4.5. In Vivo Studies

All animal experiments were conducted in accordance with the German Animal Welfare Law and approved by Berlin authorities (Landesamt für Arbeitsschutz, Gesundheitsschutz und technische Sicherheit Berlin, LAGetSi; code number A0378/12). When a body weight loss of >10% was observed, treatment was ceased until recovery. In the systemic models, mice were sacrificed individually when signs of leukemia were observed (>20–30% body weight loss, hind leg paralysis, or general deterioration of health status). The molecular alterations of the tested in vivo models are described in Supplementary Table S1.

For the systemic MOLM-13 and MV-4-11 models, female CB17-SCID (Janvier Labs, Le Genest-Saint-Isle, France) or NOD SCID (Taconic, Køge, Denmark) mice were injected intravenously (i.v.) with 200 µL of 7.5 × 10^6^ or 5 × 10^6^ cancer cells in 0.9% NaCl, respectively. The mice were treated with i.p. injection of IL3RA-ADC at 2.5 or 10 mg/kg once weekly (Q7D), every two weeks (Q14D), or every three weeks (Q21D). In the MOLM-13 model, treatments were started on day 10, and the study was terminated on day 124. In the MV-4-11 model, treatments were started on day 3, and the study was terminated on day 174 after tumor cell injection.

For the systemic AM7577 PDX model, female NOD/SCID mice (Shanghai Lingchang Bio-Technology Co. Ltd., LC, Shanghai, China) were injected i.v. with 100 µL of 1.4 × 10^6^ cancer cells in PBS at CrownBio (Beijing, China). The development of AML was monitored by flow cytometric analysis of the percentage of hCD45 cells in blood. On day 38 after tumor cell injection, when approximately 4% of hCD45-positive cells were present, the mice were randomized, and treatments were started. The mice were treated with i.v. injections of IL3RA-ADC at 2.5 or 10 mg/kg, Q7D or the isotype control ADC at 10 mg/kg, Q7D. In the first IL3RA-ADC treatment group, the initial dose of 2.5 mg/kg, Q7D was increased to 10 mg/kg, Q14D from the second administration onwards (indicated as 2.5 + 10 mg/kg). The study was terminated on day 104 after tumor cell injection.

For the systemic AML11655 PDX model, female CIEA NOG mice^®^ (NOD.Cg-*Prkdc^scid^ Il2rg^tm1Sug^*/JicTac, Taconic, Køge, Denmark) were injected i.v. with 400 µL of 1 × 10^7^ cancer cells in PBS at EPO Berlin-Buch GmbH (Berlin, Germany). The development of AML was monitored by the percentage of hCD45-positive cells in blood as determined by flow cytometry. Treatments were initiated on day 5 after tumor cell injection (preventive setting) or day 34 after tumor cell injection (therapeutic setting) when approximately 46% of hCD45-positive cells were detected in blood. The mice were treated with i.v. injections of IL3RA-ADC at 10 mg/kg, Q7D or the isotype control ADC at 10 mg/kg, Q7D. The study was terminated on day 118 after tumor cell injection.

For the HDLM-2 Hodgkin lymphoma model, female CB17-SCID mice (Janvier Labs, Le Genest-Saint-Isle, France) were injected subcutaneously (s.c.) with 100 µL of 1 × 10^7^ cancer cells suspended in 30% Matrigel/70% medium. Tumor volume (0.5 × length × width^2^) was determined based on twice weekly measurement of tumor area by a caliper (length and width). Treatments with IL3RA-ADC (5 or 10 mg/kg, i.p., Q7D×2) or ispinesib (10 mg/kg, i.v., Q7D×3) were started on day 49 when the tumors had reached a mean size of 100 mm^3^. The study was terminated on day 84 after tumor cell injection.

The subcutaneous MOLM-13 and MV-4-11 models as well as safety studies are described in the Supplementary Methods.

### 4.6. Statsitical Analyses

Statistical analyses were performed using R (version 3.3.2 or newer; R Foundation for Statistical Computing, Vienna, Austria) [51]. Flow cytometry and tumor volume data were analyzed using a linear model estimated with generalized least squares that included separate variance parameters for each study group or linear mixed-effects model with random intercepts and slopes for each subject. Mean comparisons between the treatment and control groups were performed using the estimated linear or linear mixed-effects model and corrected for family-wise error rate using Sidak’s method. Survival analyses were performed using the Cox proportional-hazards model and corrected for family-wise error rate using Sidak’s method. *p* values < 0.05 were considered significant.

## 5. Conclusions

The novel IL3RA-ADC with a differentiated mode-of action demonstrates selective binding and internalization to IL3RA-positive cells, which translates into selective and efficacious antitumor activity in IL3RA-positive AML and Hodgkin lymphoma models. By employing a KSP inhibitor, a stable lysine linkage between the payload and the antibody, and a legumain-cleavable linker resulting in a non-cell-permeable payload metabolite, IL3RA-ADC presents a new alternative for the treatment of IL3RA-positive malignancies. Using the KSPi as a payload in an ADC is expected to result in manageable toxicity and a broader therapeutic window compared to that reported for the systemic application of KSPi in clinical trials. Our data support further development of the IL3RA-ADC BAY-943 as an innovative approach for the treatment of patients with IL3RA-positive AML.

## Figures and Tables

**Table 1:** The antiproliferative activity of IL3RA-ADC in a panel of tumor cells.

Cell Line	Provider (Catalog No.)	Date Obtained	Date Authenticated	Origin	Anti-IL3RA ABC	IL3RA-ADC IC_50_ (M)
MV-4-11	ATCC (CRL 9591)	5/5/2008	02/05/2019	Biphenotypic B myelomonocytic leukemia	≈26,700	1.58 × 10^−10^
MOLM-13	DSMZ (ACC 554)	5/2/2008	02/05/2019	Acute myeloid leukemia	≈15,100 ^a^	6.37 × 10^−10^
HDLM-2	DSMZ (ACC 17)	19/02/2015	06/05/2015	Pleural effusion of Hodgkin lymphoma	≈74,300	1.97 × 10^−9^
L-428	origin unknown	1996	17/04/2013	Pleural effusion of Hodgkin lymphoma	≈111,300	3.97 × 10^−10^
THP-1	ATCC (TIB 202)	15/02/2006	19/03/2014	Acute monocytic leukemia	≈21,100	2.92 × 10^−9^
KG-1	ATCC (CCL 246)	28/10/2010	24/03/2011	Acute myelogenous leukemia	≈7200	8.34 × 10^−9^
HT	DSMZ (ACC567)	12/09/2013	19/03/2014	Diffuse mixed lymphoma	≈350	>3.00 × 10^−7^
NCI-H292	ATCC (CRL 1848)	13/08/2009	07/02/2012	Non-small cell lung cancer	≈500	>3.00 × 10^−7^
HBL-1	Charité (Prof. Lenz)	15/04/2011	03/11/2017	Diffuse B cell lymphoma	n.d.	n.d.
Kasumi-3	DSMZ (ACC 714)	20/04/2017	05/09/2017	Acute myeloid leukemia	23,500	6.89 × 10^−9 b^
Rec-1	ATCC (CRL-3004)	24/02/2014	23/08/2018	Mantle cell lymphoma	n.d.	1.03 × 10^−7^
OVCAR-8	NCI (NCI-60 panel)	20/10/2008	02/05/2019	Ovarian cancer	n.d.	1.47 × 10^−7^
MDA-MB-231	ATCC HTB-26	05/04/2006	15/10/2019	Breast cancer	≈890	>3.00 × 10^−7^
Ramos	ATCC CRL 1596	08/03/2011	06/0572015	Burkitt’s lymphoma	n.d. ^a^	n.d.

In vitro cytotoxicity (CellTiter-Glo^®^, Promega) of the IL3RA-ADC BAY-943 in cancer cell lines with different levels of anti-IL3RA antibody bound per cell (ABC) as determined by quantitative flow cytometry. The mean IC_50_ values from up to six individual assays are shown. n.d., not determined. ^a^ IL3RA expression analyzed by IHC on paraffin-embedded cell pellets; ^b^ IC_50_ determined at 144 h (at 72 h for the other cell lines).

**Table 2:** The selectivity of IL3RA-ADC compared to the isotype control ADC.

		Cytotoxicity, IC_50_ (M)				
Compound	DAR	MV-4-11 (IL3RA ≈26,700)	MOLM-13 (IL3RA ≈15,100)	HDLM-2 (IL3RA ≈74,300)	THP-1 (IL3RA ≈21,100)	NCI-H292 (IL3RA ≈500)
IL3RA-ADC	6.3	1.58 × 10^−10^	6.37 × 10^−10^	1.29 × 10^−9^	2.92 × 10^−9^	>3.00 × 10^−7^
Control ADC	7	>3.00 × 10^−7^	2.18 × 10^−9^	1.52 × 10^−7^	1.48 × 10^−8^	2.12 × 10^−7^
IL3RA-Ab	n.a.	>3.00 × 10^−7^	>3.00 × 10^−7^	>3.00 × 10^−7^	>3.00 × 10^−7^	>3.00 × 10^−7^
KSPi SMOL	n.a.	9.05 × 10^−11^	8.95 × 10^−11^	1.00 × 10^−10^	3.07 × 10^−10^	2.16 × 10^−10^

In vitro cytotoxicity (CellTiter-Glo^®^, Promega) of the IL3RA-ADC BAY-943, isotype control ADC BAY-229, IL3RA antibody TPP-9476, and small molecule KSP inhibitor BAY-331 in the IL3RA-positive AML cell lines MV-4-11, MOLM-13, HDLM-2, THP-1 and in the IL3RA-low expressing NSCLC cell line NCI-H292 after 72 h incubation time. Anti-IL3RA ABC levels as determined by quantitative flow cytometry are indicated in the parentheses after each cell line. Ab, antibody; ADC, antibody–drug conjugate; DAR, drug-to-antibody ratio; n.a., not applicable; NSCLC, non-small-cell lung carcinoma; SMOL, small molecule.

## Supplementary Materials

The following are available online at https://www.mdpi.com/2072-6694/12/11/3464/s1, Figure S1: Drug-to-antibody ratio (DAR) of the IL3RA-ADC BAY-943, Figure S2: Apoptotic activity of the IL3RA-ADC BAY-943 in IL3RA-positive cells in vitro, Figure S3: Body weights in the systemic MOLM-13 and MV-4-11 leukemia xenograft models, Figure S4: Antitumor efficacy of the IL3RA-ADC BAY-943 in the subcutaneous MOLM-13 human AML and MV-4-11 human biphenotypic leukemia xenograft models, Figure S5: IL3RA expression in the patient-derived AML11655 and AM7577 AML xenografts models and cell line-derived AML and HL models, Figure S6: Time course of relative body weight changes in mouse models, Table S1: Characteristics of the in vivo mouse xenograft models.

## Abbreviations

Ab	antibody
ABC	antibodies bound per cell
ADC	antibody–drug conjugate
AML	acute myeloid leukemia
BPDCN	blastic plasmacytoid dendritic cell neoplasm
CAR-T	chimeric antigen receptor T cell
cHL	classical Hodgkin lymphoma
DAR	drug-to-antibody ratio
DLBCL	diffuse large B-cell lymphoma
EC_50_	half-maximal effective concentration
FACS	fluorescence-activated cell sorting
FCS	fetal calf serum
hCD45	human CD45
hIL3RA	human IL3RA
HL	Hodgkin lymphoma
IC_50_	half-maximal inhibitory concentration
IGN	indolinobenzodiazepine pseudodimer
IL-3	interleukin 3
IL3RA	interleukin 3 receptor subunit alpha
IL3RA-Ab	IL3RA-targeting antibody
i.p.	intraperitoneal(ly)
i.v.	intravenous(ly)
KD	dissociation constant
KSP	kinesin spindle protein
KSPi	kinesin spindle protein inhibitor
MDS	myelodysplastic syndrome
MFI	median fluorescence intensity
MST	median survival time
Papp	apparent permeability
PBD	pyrrolobenzodiazepine
PBS	phosphate-buffered saline
pDCs	plasmacytoid dendritic cells
PDX	patient-derived xenograft
P-gP	P-glycoprotein
Q7D	once weekly
Q14D	every two weeks
Q21D	every three weeks
rrMM	relapsed refractory multiple myeloma
s.c.	subcutaneous(ly)
siRNA	small interfering RNA
SPR	surface plasmon resonance
