# Supplementary material for: IL3RA-Targeting Antibody–Drug Conjugate BAY-943 with a Kinesin Spindle Protein Inhibitor Payload Shows Efficacy in Preclinical Models of Hematologic Malignancies
Source: Cancers (Basel). 2020 Nov 20;12(11):3464. doi: 10.3390/cancers12113464 (PMC7709048; doi:10.3390/cancers12113464)
Supplement: Supplementary file 1 [file cancers-12-03464-s001.zip › Kirchhoff et al_Appendix B_Suppl figs and tables_201113.docx]

Article

IL3RA-Targeting Antibody-Drug Conjugate BAY-943 with a Kinesin Spindle Protein Inhibitor Payload Shows Efficacy in Preclinical Models of Hematologic Malignancies

Dennis Kirchhoff ^1,^*, Beatrix Stelte-Ludwig ^2^, Hans-Georg Lerchen ^2^, Antje Margret Wengner ^1^, Oliver von Ahsen ^1^, Pascale Buchmann ^2^, Stephan Märsch ^2^, Christoph Mahlert ^2^, Simone Greven ^2^, Lisa Dietz ^2^, Michael Erkelenz ^1^, Ruprecht Zierz ^1^, Sandra Johanssen ^1^, Dominik Mumberg ^1^ and Anette Sommer ^1^

^1^ Bayer AG, Pharmaceuticals, Research & Development, 13342 Berlin, Germany; antje.wengner@bayer.com (A.M.W.); oliver.vonahsen@bayer.com (O.v.A.); michael.erkelenz@bayer.com (M.E.); ruprecht.zierz@bayer.com (R.Z.); sandra.johanssen@bayer.com (S.J.); dominik.mumberg@bayer.com (D.M.)

^2^ Bayer AG, Pharmaceuticals, Research & Development, 42096 Wuppertal, Germany; beatrix.stelte-ludwig@bayer.com (B.S.-L.); hans-georg.lerchen@bayer.com (H.-G.L.); pascale.buchmann@bayer.com (P.B.); stephanmaersch@hotmail.com (S.M.); lisa.dietz@bayer.com (L.D.); christoph.mahlert@bayer.com (C.M.); simone.greven@bayer.com (S.G.)

***** Correspondence: dennis.kirchhoff@bayer.com; Tel.: +49 30 468193479

Supplementary figures and table

|  |
| --- |

**Supplementary Figure S1.** Drug-to-antibody ratio (DAR) of the IL3RA-ADC BAY-943. (A) Distribution of payloads coupled to BAY-943 determined by HPLC coupled with ESI-Q-TOF. The proportion of unconjugated antibody was 0% and the calculated DAR was 6.3. All IL3RA-ADC batches described in this publication have comparable analytical data. (B) Determination of ADC purity by SEC-UV-MALS. Absorption of the ADC was measured at 280 nm. The dimer content of the ADC was 4.5% as indicated by a small peak at 9.3 min. (C) Stability of BAY-943 in human plasma at 37 °C. The obtained DAR values ranged from 5.6 to 5.3 over 24 h indicating that a spontaneous release of the KSPi payload from the ADC is unlikely.

|  |
| --- |

**Supplementary Figure S2.** Apoptotic activity of the IL3RA-ADC BAY-943 in IL3RA-positive cells *in vitro*. The induction of apoptosis as measured by caspase 3/7 activation in IL3RA-positive MV-4-11 human biphenotypic leukemia cells by IL3RA-ADC (A) and in IL3RA-negative MDA-MB-231 human breast cancer cells (B). RLU relative luminescence units.


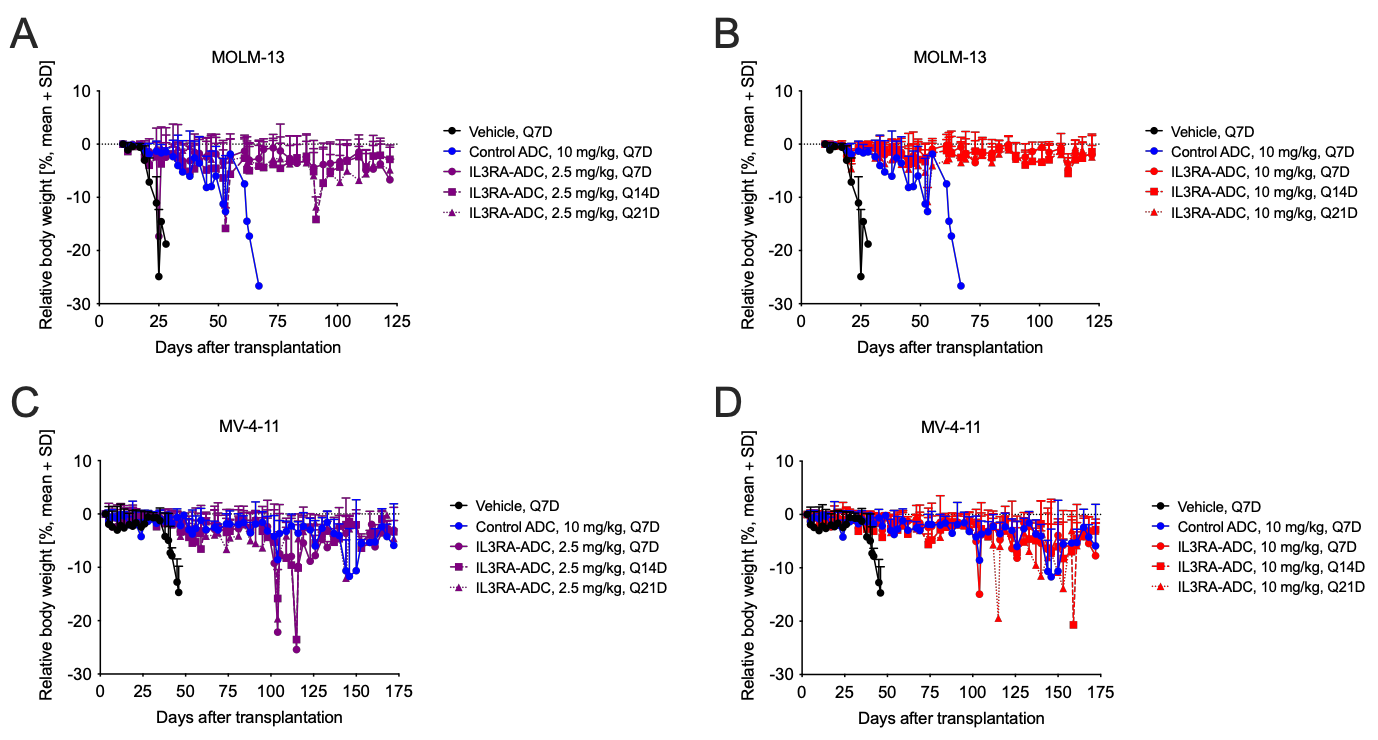


**Supplementary FigureS3.** Body weights in the systemic MOLM-13 and MV‑4-11 leukemia xenograft models. A-B. Relative body weight plots of mice transplanted with the MOLM-13 human AML cells and treated i.v. with the isotype control ADC (10 mg/kg, Q7D) or IL3RA-ADC at 2.5 mg/kg (A) or 10 mg/kg (B); Q7D, Q14D or Q21D. C-D. Relative body weight plots of mice transplanted with the MV-4-11 human biphenotypic leukemia cells and treated i.v. with the isotype control ADC (10 mg/kg, Q7D) or IL3RA-ADC at 2.5 mg/kg (C) or 10 mg/kg (D); Q7D, Q14D or Q21D.

|  |
| --- |

**Supplementary Figure S4.** Antitumor efficacy of the IL3RA-ADC BAY-943 in the subcutaneous MOLM-13 human AML and MV-4-11 human biphenotypic leukemia xenograft models. Mice were inoculated s.c. with 2x10^6^ MOLM-13 or 5x10^6^ MV-4-11 cells and treatments were initiated on day 3. (A) Growth curves of MOLM-13 tumors treated with the isotype control ADC (Q7Dx2, i.p.), IL3RA-ADC (Q4Dx6 for 2.5 mg/kg, Q7Dx2 for 5 and 10 mg/kg, i.p.) or cytarabine (QDx5, i.p.). Arrows indicate the time of treatment. (B) Tumor volumes in IL3RA-ADC-treated mice described in panel A on day 21. (C) Growth curves of MV-4-11 tumors treated with the isotype control ADC (5 mg/kg, Q7Dx2, i.p.), IL3RA Ab (5 mg/kg, Q7Dx2, i.p.), cytarabine (50 mg/kg, QDx5, i.p.) or IL3RA-ADC (0.6, 1.25, 2.5, 5, or 10 mg/kg, Q7Dx2, i.p.). Arrows indicate the time of treatment. (D) Tumor volumes in mice described in panel C on day 18. Statistical analyses were performed on day 21 (MOLM-13) or 18 (MV-4-11) using a linear mixed-effects model with random intercepts and slopes for each subject (n = 8). Mean comparisons between the treatment and control groups were performed using the estimated linear mixed-effects model and corrected for family-wise error rate using Sidak’s method. Asterisks and hashtags indicate statistical significance in comparison to the vehicle (^***^p < 0.001) or isotype control ADC (^###^p < 0.001).


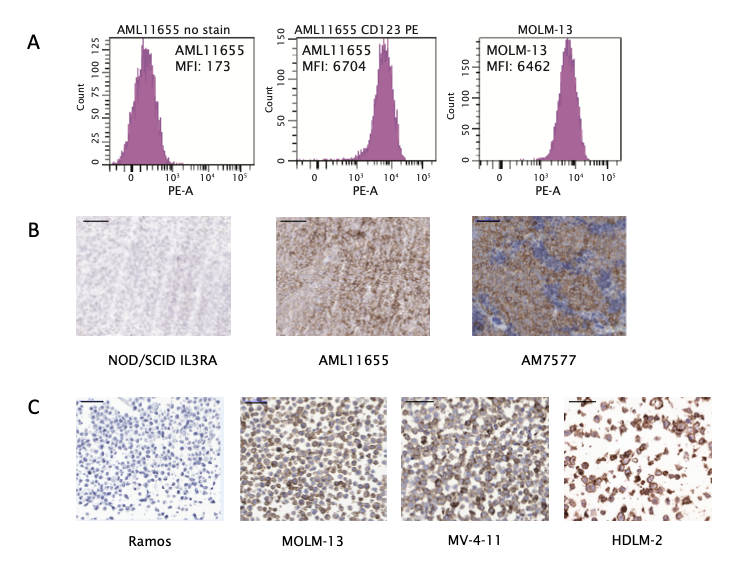


**Supplementary Figure S5.** IL3RA expression in the patient-derived AML11655 and AM7577 AML xenografts models and cell line-derived AML and HL models described in this manuscript. (A) IL3RA expression in the AML11655 patient-derived tumor cells as detected by flow cytometry. “No stain” indicates that no primary IL3RA antibody was added, only the secondary antibody. MFI: Mean fluorescence intensity. IL3RA-positive MOLM-13 cells were analyzed for comparison. (B) IL3RA expression in the spleens of systemic AML11655 or AM7577 xenografts as determined by IHC. The spleen of a tumor free NOD/SCID mouse represents an IL3RA-negative control. (C) IL3RA expression in MOLM-13, MV-4-11 and HDLM-2 cells (IL3RA-positive) and Ramos cells (IL3RA-negative). Bar = 50 μm.


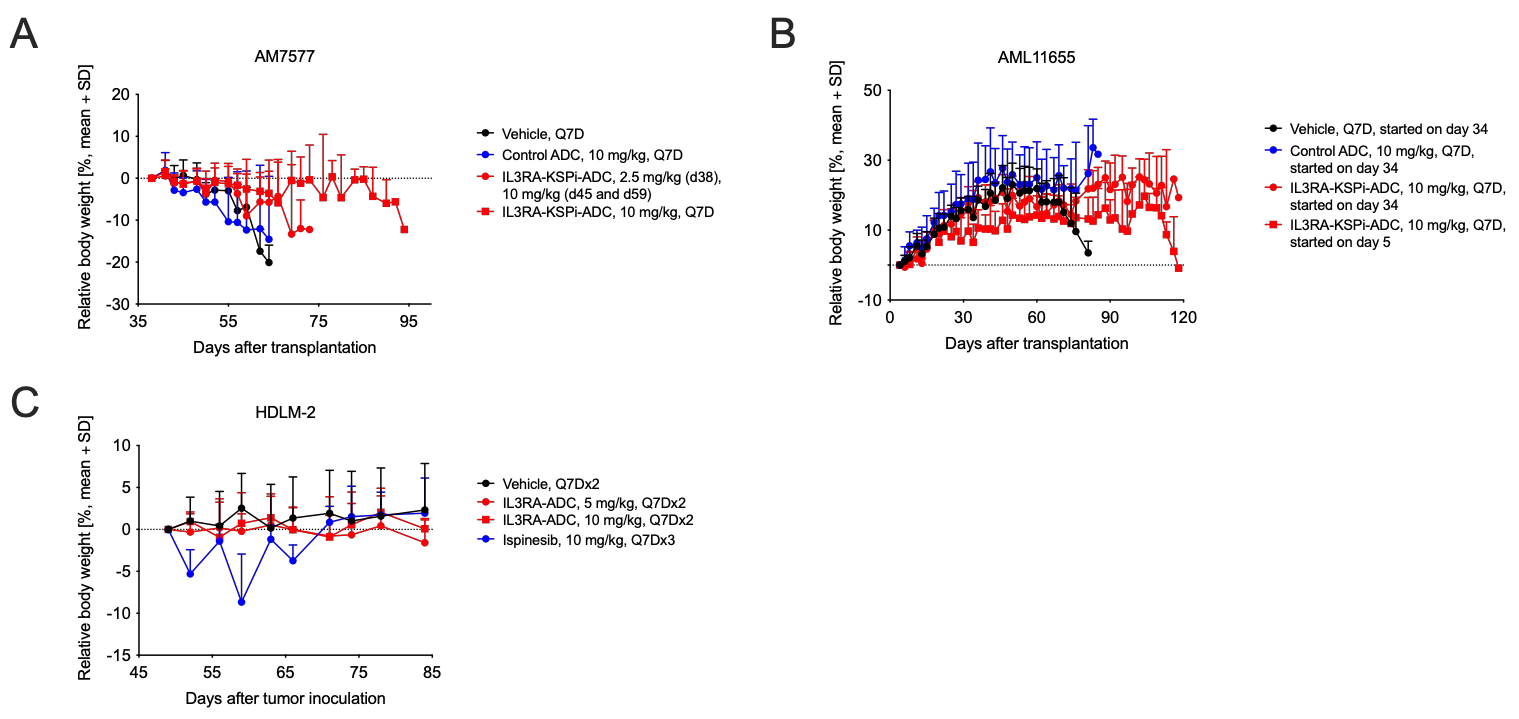


**Supplementary Figure S6.** Time course of relative body weight changes in mouse models described in (A) Figure 3A-B, (B) Figure 3C-D, and (C) Figure 4.

**Table 1.** Characteristics of the in vivo mouse xenograft models.

| **Mouse model** | **Indication** | **Ethnicity** | **Gender** | **Age** | **Molecular alterations** | **Pre-treatment / establishment history** | **Literature** |
| --- | --- | --- | --- | --- | --- | --- | --- |
| MOLM-13 (CDX) | Acute monoblastic leukemia (M5a) | Asian (Japan) | Male | 20 | Karyotype: 47, XY, +8, ins t(9;11)(p22;q23); MLL-AF9 gene fusion | MDS (RAEB) – AML-M5a; at relapse, after chemotherapy (established in 1995) |  |
| MV-4-11 (CDX) | Biphenotypic B myelomonocytic leukemia (M5) | Caucasian | Male | 10 | Karyotype: t(4;11)(q21;q23) MLL-AF4; FLT3-ITD | Established at diagnosis |  |
| HDLM-2 (CDX) | Hodgkin lymphoma | Caucasian | Male | 74 | Inactivation of A20 (TNFAIP3) | Established from the pleural effusion of HL pat. (nodular sclerosing, stage IV) in 1982 | Schmitz *et al*. TNFAIP3 (A20) is a tumor suppressor gene in Hodgkin lymphoma and primary mediastinal B cell lymphoma. J Exp Med 2009. |
| AM7577 (PDX) | Acute monocytic leukemia (M5) | Asian | Male | 68 | Normal karyotype, Mutations: FLT3-ITD (+); DNMT3A (R882H); IDH2 (R140Q), NPM1 and CEBPA insertion (SNP) | Patient received several cycles of ECAG [etoposide in combination with low-dose CAG (cytarabine, aclarubicin, G-CSF)], CR after 1st cycle, relapse, AML sample for generation of PDX model was obtained after relapse | Liu *et al*. A unique leukemia mouse model established from AML patient with IDH2 R140Q and FLT3-ITD mutations among other common AML mutations.  ASH Annual Meeting 2012.  Liu *et al*. Modeling anti-leukemic therapy by patient derived AML xenografts with distinct phenotypes/genotypes. AACR Annual Meeting 2014. |
| AM11655 (PDX) | Acute myeloblastic leukemia (M1) | Caucasian | Male | 43 | Sequence variations: IDH1, NPM1 | Pre-treatment history unknown |  |

CDX, cell line-derived xenograft; PDX, patient-derived xenograft.
