# Supplementary material for: IL3RA-Targeting Antibody–Drug Conjugate BAY-943 with a Kinesin Spindle Protein Inhibitor Payload Shows Efficacy in Preclinical Models of Hematologic Malignancies
Source: Cancers (Basel). 2020 Nov 20;12(11):3464. doi: 10.3390/cancers12113464 (PMC7709048; doi:10.3390/cancers12113464)
Supplement: Supplementary file 1 [file cancers-12-03464-s001.zip › Kirchhoff et al_Appendix A_Suppl methods.docx]

Article

IL3RA-Targeting Antibody-Drug Conjugate BAY-943 with a Kinesin Spindle Protein Inhibitor Payload Shows Efficacy in Preclinical Models of Hematologic Malignancies

Dennis Kirchhoff ^1,^*, Beatrix Stelte-Ludwig ^2^, Hans-Georg Lerchen ^2^, Antje Margret Wengner ^1^, Oliver von Ahsen ^1^, Pascale Buchmann ^2^, Stephan Märsch ^2^, Christoph Mahlert ^2^, Simone Greven ^2^, Lisa Dietz ^2^, Michael Erkelenz ^1^, Ruprecht Zierz ^1^, Sandra Johanssen ^1^, Dominik Mumberg ^1^ and Anette Sommer ^1^

^1^ Bayer AG, Pharmaceuticals, Research & Development, 13342 Berlin, Germany; antje.wengner@bayer.com (A.M.W.); oliver.vonahsen@bayer.com (O.v.A.); michael.erkelenz@bayer.com (M.E.); ruprecht.zierz@bayer.com (R.Z.); sandra.johanssen@bayer.com (S.J.); dominik.mumberg@bayer.com (D.M.)

^2^ Bayer AG, Pharmaceuticals, Research & Development, 42096 Wuppertal, Germany; beatrix.stelte-ludwig@bayer.com (B.S.-L.); hans-georg.lerchen@bayer.com (H.-G.L.); pascale.buchmann@bayer.com (P.B.); stephanmaersch@hotmail.com (S.M.); lisa.dietz@bayer.com (L.D.); christoph.mahlert@bayer.com (C.M.); simone.greven@bayer.com (S.G.)

***** Correspondence: dennis.kirchhoff@bayer.com; Tel.: +49 30 468193479

Supplementary Methods

Tryptic/pepsin peptide mapping for identification of TPP-9476

For peptide mapping, the antibody was diluted in 20 mM sodium phosphate pH 6.5 to a concentration of 20 pmol/µL and denatured by 5 M guanidine hydrochloride (in 50 mM triethylammonium hydrogencarbonate buffer (TEAHC) and boiling at 60 °C for 10 min. To reduce the protein, 255 mM dithiothreitol (DTT) in water was added and heated at 60 °C for 20 min. To prevent reformation of disulfide bonds, 535 mM iodine acetamide (IAA) solution in water and 50 mM TEAHC were added and the sample was incubated under gentle shaking (Thermomixer) at 45 °C for 20 min. Next, the free iodine acetamide was captured by incubating the sample with 255 mM DTT under gentle shaking at 45 °C for 20 min. The sample was divided into two parts: Part I was digested by trypsine at an enzyme:protein ratio of 1:20; Part II was acidified with 1 M hydrochloride and digested by pepsin at an enzyme:protein ratio of 1:50. Part I and Part II samples were incubated under gentle shaking at 37 °C for 24 h. The Part I sample was acidified with 10% formic acid at room temperature.

For separation of digested peptides and MS analysis for sequence coverage, aliquots of Part I and Part II samples were each diluted with a solution consisting of 0.1% formic acid, 5% acetonitrile, 95% water. Acquity UPLC-BEH C18 and VanGuard Acquity UPLC-BEH C18 columns were used for separation and analyzed by HPLC ESI-Q-TOF mass spectrometry using a Synapt G2-S HDMS ESI-Q-TOF equipped with MassLynx software and ProteinLynx Global Server as data preparation tools for Mascot database search. Combined sequence coverage was generated based on comparison of the acquired MS/MS masses and the theoretical tryptic or pepsin peptide masses based on the Mascot database search algorithm.

Binding affinity of IL3RA-Ab

To assess the selectivity of the anti-IL3RA antibody TPP-9476 (IL3RA-Ab), its binding to IL3RA was determined by surface plasmon resonance (SPR) and flow cytometry. IgGs were captured via an amine-coupled anti-human IL3RA-Fc capture antibody and analyzed using a Biacore Sensor Chip CM5 (GE Healthcare). Antigen binding was tested using antigen concentrations of 1.56 - 200 nM at 25 °C, and the KD values were subsequently evaluated with a 1:1 Langmuir binding model. For flow cytometry, MOLM-13, MV-4-11 and KG-1 cells (5.55 x 10^5^/well) were incubated with the primary anti-ILR3A antibody TPP-9476 (3.3 x 10^-7^ – 4 x 10^-12^ M) on ice for 1 h. The reaction was stopped by adding ice-cold fluorescence-activated cell sorting (FACS) buffer consisting of phosphate-buffered saline (PBS) and 3% fetal bovine serum (FBS), and the samples were centrifuged at 400 x g and 4 °C for 5 min. The cell pellet was washed once with FACS buffer prior to incubation with the secondary antibody (goat IgG anti-human IgG (F(ab’)2)-Alexa Fluor 488, cat. # 109-545-097 Dianova / Jackson ImmunoResearch, 1:200 dilution) for 1 h, followed by centrifugation and washing. The final cell pellet was resuspended in ice-cold FACS buffer including propidium iodine as live/dead cell-discriminating stain.

Determination of the drug-to-antibody ratio (DAR)

The number of drug molecules per antibody (drug-to-antibody ratio, DAR) of the IL3RA-ADC BAY-943 was determined after standard native deglycosylation using a combination of HPLC and ESI-Q-TOF. Due to kinesin spindle protein inhibitor (KSPi) toxophore coupling to different lysine residues, a distribution of masses was observed (Supplementary Fig. A1). Additional masses were detected due to toxophore fragmentation (highlighted as D*) during electrospray ionization (ESI) within the MS system. The peak area of the detected fragments was added to the peak area of the following toxophore species and included in DAR calculation. The DAR was obtained from the sum of the toxophore number weighted peak area divided by the sum of unweighted peak area.

Homogeneity and purity assessment of IL3RA-ADC

The homogeneity and purity of the ADC was assessed using size exclusion chromatography (SEC) with UV coupled with multi-angle light scattering (MALS). SEC-UV-MALS analysis was carried out on an Agilent 1200 HPLC system monitored at 280 nm coupled to a miniDAWN Treos light scattering detector and an Optilab rEX interferometric refractometer (Wyatt Technology Corporation). A Superdex200 10/300 GL column (GE Healthcare) was operated with an isocratic gradient using PBS at a flow rate of 0.5 mL/min at room temperature. The dimer content was determined from the integration of UV peaks of monomeric and dimeric ADCs

Apoptotic activity of IL3RA-ADC

The apoptotic activity of IL3RA-ADC was assessed using the Caspase-Glo 3/7 assay (Promega). Cells were plated in 96-well plates (MV-4-11: 4,000 cells/well; MDA-MB-231 4,000 cells/well) and incubated at 37 °C, 5% CO_2_ for 24 h. After incubation, cells were either left untreated or treated with the respective compounds at 3 x 10^-11^ M – 3 x 10^-7^ M for 48 h. Thereafter, Caspase-Glo 3/7 reagent was added to each well and the plates were incubated at room temperature for 45 min. The luminescence was recorded for 0.2 s/well on a Victor V instrument (Perkin Elmer). The EC_50_ values were calculated with the internal BELLA Dose Range Concentration (DRC) software.

IL3RA expression

IL3RA expression was determined in AML11655 patient-derived AML cells by flow cytometry and in the spleen of AM7577 AML cell-bearing mice as well as in paraffin-embedded MOLM-13, MV4-11, HDLM-2 and Ramos cells by immunohistochemistry (IHC) using an anti-CD123 antibody (Leica Biosystems).

For IHC, 3 µm sections of mouse spleen or cancer cell pellets as FFPE samples were analyzed using standard procedures. In brief, antigen retrieval was performed in target retrieval solution [Dako S2367 (all Dako reagents now provided by Agilent, Santa Clara, CA, USA)] in pH 9 for 17 min in a steam cooking device. Endogenous peroxidase activity was blocked with peroxidase-blocking solution (Dako S202386) for 15 min at room temperature. IL3RA protein was stained using the primary mouse anti-CD123 antibody clone BR4MS (Leica Biosystems) at 2 µg/L final concentration in antibody diluent (Dako S2022) for 2 hours, followed by application of the DAKO EnVision system (anti-mouse; Dako K4006) for 30 min and addition of the DAB chromophore for 10 min. Incubation with antibodies was performed in a humid chamber at room temperature.

Caco-2 permeability assay

The permeability of the active toxophore metabolite BAY-716 was tested in Caco-2 cell monolayers according to Artursson and Karlsson, 1991 (1). Briefly, Caco-2 cells (ACC 169, DSMZ, Braunschweig, Germany) were seeded and allowed to grow for 14-16 days. For permeability studies, the test compounds were dissolved in DMSO and diluted to the final test concentration of 2 µM with transport buffer containing Hanks’ buffered salt solution (Gibco/Invitrogen) supplemented with glucose (final concentration 19.9 mM) and HEPES (final concentration 9.8 mM). For determination of the apical to basolateral permeability (Papp A–B), BAY-716 solution was added to the apical side of the cell monolayer and transport buffer to the basolateral side of the monolayer. Samples were taken from the donor compartment at the beginning of the experiment to confirm mass balance. After an incubation at 37 °C for 2 h, samples were taken from both compartments. The samples were analyzed by LC-MS and the apparent permeability coefficients were calculated. The efflux ratio was calculated as Papp B–A/Papp A–B. Lucifer yellow permeability was assayed for each cell monolayer to ensure cell monolayer integrity, and the permeability of atenolol (low permeability marker) and sulfasalazine (marker for active excretion) was determined for each batch as a quality control.

Determination of stability of BAY-943 in human plasma

For the generation of IL3RA-coated magnetic beads, IL3RA (R&D Systems) was biotinylated with EZ-Link Sulfo-NHS-LC-Biotin (Thermo Fisher Scientific). IL3RA at 10 µM was incubated in the presence of 310 µM EZ-Link Sulfo-NHS-LC-Biotin at room temperature for 2.5 h and then kept at 4 °C overnight. Unreacted biotin was removed with Zeba Spin Desalting Columns (Thermo Fisher Scientific) according to the manufacturer’s manual. A suspension of Strep-Tactin® XT coated magnetic beads, 5 % (v/v) (IBA Life Sciences) was washed three times with the manufacturer’s buffer W (100 mM Tris/HCl, 150 mM NaCl, 1 mM EDTA, pH 8.0). The beads in buffer W were mixed with the biotinylated IL3RA (at ~8.3 µM) and the mixture was incubated at room temperature for 3 h. Subsequently, the beads were washed four times in buffer W and finally stored in buffer W at a concentration of 8.4% (v/v).

To measure the stability of the IL3RA-ADC BAY-943 in human plasma, the ADC was treated with PNGase to remove glycans prior to stability testing. To this end, ADC was digested with 1,200 U PNGase F (Promega) in 25 mM ammonium carbonate buffer. Complete removal of glycans was confirmed by mass spectrometry. The deglycosylated ADC was mixed with human plasma to a final concentration of 167 ng/µL ADC in 97% plasma. The plasma was incubated at 37 °C and samples were taken at 0, 0.25, 2, 4, and 24 h and subjected to immunoprecipitation. The samples were diluted (3:100) with 50 mM Tris/HCl, 150 mM NaCl, 0.05% Tween 20, pH 7.6. Affinity capture was performed by adding 20 µL of IL3RA-coated magnetic beads suspension (8.4% (v/v beads wet volume) to a final concentration of 0.16% (v/v). The mixture was subsequently incubated overnight at 4 °C under rotation mixing. Magnetic beads were separated and washed with different buffer/detergent solutions: 1) 50 mM Tris/HCL, 150 mM NaCl, 0.05% Tween 20; 2) 100 mM Tris/HCl, 150 mM NaCl, 1 mM EDTA; 3) deionized water. ADCs were eluted in formic acid (2%) and incubated with shaking at room temperature for 30 min. The eluate was filtrated (Millex PTFE-Filter Unit; 0.20 µm; Merck) and thereafter, subjected to LC-MS analysis using the Easy nanoLC 1200 system coupled to a Q Exactive plus mass spectrometer (Thermo Fisher Scientific). The DAR was calculated as described above with the exception that additional masses caused by toxophore fragmentation were not taken into account.

Subcutaneous leukemia models

For the subcutaneous leukemia models, 2 x 10^6^ MOLM-13 human AML or 5 x 10^6^ MV-4-11 human biphenotypic leukemia cells were suspended in 100% Matrigel® and inoculated subcutaneously (s.c.) by injection of 100 µL cell suspension into the flank of female NMRI *nu/nu* mice (Janvier Labs, France). Treatments with intraperitoneal (i.p.) injections of IL3RA-ADC at 0.6 – 10 mg/kg, isotype control ADC at 10 mg/kg, IL3RA-Ab at 5 mg/kg or cytarabine at 50 mg/kg were started on day 3 after tumor cell inoculation. Tumor volume (0.5 x length x width^2^) was determined based on tumor area measurement by caliper (length and width). Tumor area and body weight were determined 2 – 3 times per week. Changes in body weight throughout the study compared to initial body weight at treatment start was considered the measure of treatment-related toxicity (>10% = critical, treatment on hold until recovery; >20% = toxic, termination). The mice were euthanized when showing signs of toxicity (>20% body weight loss), or when tumors reached a maximum size of 1500 mm^3^. The MOLM-13 study was terminated on day 49, the MV-4-11 study on day 60 after tumor cell inoculation.

Safety studies

The safety profile of IL3RA-ADC was evaluated in naïve cynomolgus monkeys. Young female (F) or male (M) monkeys were given a single, slow intravenous (i.v.) injection of IL3RA-ADC at 10 mg/kg (n=2F) or at 20 mg/kg (n=2F) and of isotype control ADC at 10 mg/kg (n=1F, 1M). Peripheral blood was analyzed by flow cytometry before treatment (0 h) and at several time points (24 h, day 8, day 15, and day 21). The small populations of basophils and plasmacytoid dendritic cells (pDCs) expressing IL3RA were anazlyzed. In addition, bone marrow cells were analyzed cytometrically to determine the potential impact of the treatments on the hematopoietic stem cells containing the CD34^+^Lin^-^ cell population.

In a repeated-dose safety study, cynomolgus monkeys were given i.v. injection of IL3RA-ADC at 2.5, 5, or 10 mg/kg (1F and 1M/treatment) or isotype control ADC at 8 mg/kg every 3 weeks for 3 cycles. For toxicokinetic assessment, blood samples were collected 1 h, 6 h, 24 h, 48 h, 96 h, 168 h, and 480 h after the first dosing on day 1 and 1 h, 6 h, 24 h, 48 h, 96 h, and 168 h after the third dosing on day 43. For flow cytometry analysis, blood samples were collected before and 1, 2, 8, 21, 29, 43, 44, and 50 days after the first dosing. Toxicological assessments also included the evaluation of body weight, food/water intake, daily clinical observation, as well as macroscopic and microscopic pathology. The animals were euthanized, organs were weighed, and tissues were collected at necropsy.

The effect of BAY-716, the non-cell permeable active payload metabolite of IL3RA-ADC, was analyzed after a single i.v. dose of 0.25 mg/kg in male rats (n=6) and after i.v. injection every 3 weeks for 3 cycles. Besides standard toxicological assessment, toxicokinetic assessment and histopathology were also performed.

References

1. Artursson P, Karlsson J. Correlation between oral drug absorption in humans and apparent drug permeability coefficients in human intestinal epithelial (Caco-2) cells. Biochem Biophys Res Commun **1991**;175(3):880-5 doi 10.1016/0006-291x(91)91647-u.

| 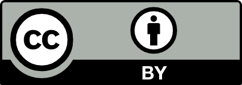 | © 2020 by the authors. Submitted for possible open access publication under the terms and conditions of the Creative Commons Attribution (CC BY) license (http://creativecommons.org/licenses/by/4.0/). |
| --- | --- |
